# Diurnal change of retinal vessel density related to hemodynamic variation in treatment-naïve low-teens normal-tension glaucoma

**DOI:** 10.1038/s41598-023-37214-w

**Published:** 2023-06-30

**Authors:** Sung Uk Baek, Soonil Kwon, Young Kook Kim, Jin Wook Jeoung, Ki Ho Park

**Affiliations:** 1 Department of Ophthalmology, Hallym University College of Medicine, Anyang, Republic of Korea; 2 Department of Ophthalmology, Hallym University Sacred Heart Hospital, Anyang, Republic of Korea; 3 Department of Ophthalmology, Seoul National University College of Medicine, Seoul, Republic of Korea; 4 Department of Ophthalmology, Seoul National University Hospital, Seoul National University College of Medicine, 101 Daehak-Ro, Jongno-Gu, Seoul, 03080 Republic of Korea

**Keywords:** Optic nerve diseases, Glaucoma

## Abstract

This study undertook to investigate the diurnal variation of optical coherence tomography angiography (OCTA) -derived retinal vessel density (RVD) in glaucoma patients with low baseline intraocular pressure (IOP). A prospective evaluation was performed on low-teens normal-tension glaucoma (low-teens NTG) patients with pre-treatment IOP < 15 mmHg and 32 healthy subjects. Superficial peripapillary and macular RVD by OCTA, IOP, and systemic blood pressure (BP) were all measured four times per day (from 9:00 a.m. to 6:00 p.m.). In the low-teens NTG group, the magnitude of diurnal changes in peripapillary RVD and macular RVD were greater than those in the healthy group. Diurnal variations of diastolic BP (DBP) and mean ocular perfusion pressure (MOPP) also were greater in the low-teens NTG group. As for the patterns of diurnal RVD change, the inferior and temporal sections of macular RVD showed significant differences between the two groups. Diurnal changes of RVD and MOPP and were greater than those in healthy eyes. The macular RVD and MOPP showed different diurnal patterns between the two groups. From these findings, OCTA-derived RVD variation could be related to hemodynamic variability in low-teens NTG.

## Introduction

Intraocular pressure (IOP) being, at present, the most significant risk factor and sole reliable therapeutic target for the currently available treatments all aim to reduce IOP by pharmacologic, laser, or surgical intervention^1^. However, certain patients suffer from glaucoma even though they have relatively low IOP. In such cases, involvement of risk factors other than elevated IOP might be central^2^. The vascular theory, meanwhile, ascribes the pathogenesis of glaucomatous optic neuropathy (GON) to reduced perfusion pressure and faulty vascular autoregulation^2^. Compromised optic nerve perfusion likely plays a role in GON, and it may have a more central role in the glaucoma setting despite low or normal IOP^3^.

A recent study conducted by the present authors showed that fluctuation of diastolic blood pressure (DBP) and diurnal IOP variations were significantly associated with greater probability of disease progression in low-teens NTG eyes^4^. Based on these results, we could speculate that hemodynamic variability in low-teens NTG may play an important role in disease course. Meanwhile, advances in imaging devices such as optical coherence tomography (OCT) and OCT angiography (OCTA) have allowed for detailed visualization of superficial and deep vasculature as well as optic nerve head (ONH) structural characteristics^5,6^. In an earlier study, the present authors, having analyzed diurnal variation in choroidal thickness using OCT, reported that diurnal variation of choroidal thickness representing the deep vasculature was significant in patients with primary open-angle glaucoma (POAG)^7^. Subsequently, others noted that in open-angle glaucoma (OAG) eyes, diurnal change of superficial retinal vessel density (RVD) was significantly greater when compared with healthy eyes and that such change was correlated with mean ocular perfusion pressure (MOPP) change^8^. All of these findings suggest that diurnal RVD changes might reflect clinically important hemodynamic variation thought to be peasant in OAG^9^.

Those studies, however, had enrolled patients who were taking ocular hypotensive medication. Also, those participants usually were POAG patients with higher baseline IOP. In other words, our previous studies may not have fully represented actual hemodynamic variability in normal-tension glaucoma (NTG) patients, especially those not on ocular hypotension medication.

The present study therefore undertook to investigate diurnal variation of RVD and the related ocular and hemodynamic factors including IOP, blood pressure (BP), and MOPP in patients diagnosed with low-teens NTG compared with healthy subjects. The aim was to elucidate the pattern and extent of hemodynamic instability observable among patients with low-teens NTG.

## Results

The baseline characteristics of the study population are presented in Table 1. There were no significant inter-group differences in age, sex, body-mass index (BMI), diabetes mellitus, hypertension, spherical equivalent, axial length, central corneal thickness, or baseline IOP (all *P* > 0.05). There also were no differences in number or type of antihypertensive treatment. As expected, low-teens NTG eyes had a higher vertical cup-to-disc ratio, a thinner average retinal nerve fiber layer (RNFL) thickness and worse visual field results than did the healthy eyes (all *P* < 0.001).

**Table 1:** Demographics and clinical characteristics of healthy subjects and low-teens NTG.

	Healthy group (N = 32)	Low-teens NTG (N = 35)	*P*-value
Age (years)	59.6 ± 13.5	58.7 ± 14.2	0.655*
Sex (Male : Female)	15 : 17	15 : 19	0.266^†^
Body-mass index (Kg/m^2^)	23.97 ± 2.51	23.63 ± 3.07	0.836*
Diabetes mellitus, n (%)	6 (18.8)	7 (20.0)	0.878^†^
Hypertension, n (%)	9 (28.1)	11 (31.4)	0.945^†^
No. of antihypertensive medications, n			
No medication	0	1	0.534^†^
1 medication	3	4	
≥ 2 medications	6	6	
Antihypertensive medication type			
ACEIs	4	3	0.224^†^
Angiotensin II receptor blocker	2	2	
CCBs	5	5	
Diuretics	2	3	
BRBs	4	5	
Spherical equivalent (Diopters)	-1.05 ± 2.32	-1.75 ± 3.07	0.193*
Axial length (mm)	23.96 ± 1.18	24.03 ± 1.23	0.597*
Central corneal thickness (µm)	534.46 ± 26.76	540.14 ± 43.53	0.658*
Baseline IOP (mmHg)	14.2 ± 2.06	13.39 ± 1.00	0.469*
Baseline vertical cup-to-disc ratio	0.41 ± 0.14	0.64 ± 0.21	< 0.001*
Mean cpRNFL thickness (µm)	101.91 ± 11.47	78.57 ± 18.52	< 0.001*
Mean deviation (dB)	0.22 ± 5.14	-8.94 ± 8.47	< 0.001*
Glaucoma severity, n (%)		Mild:14 (40)	
		Moderate: 14 (40)	
		Advanced: 7 (20)	
		Severe: 0 (0)	

Values are presented as the mean ± standard deviation or number (%).

*Student t-test, † Chi-square test.

ACEI = angiotensin-converting enzyme inhibitor; BRB = beta receptor blocker; CCB = calcium channel blocker; cpRNFL = circumpapillary retinal nerve fiber layer.

### Assessment of repeatability of RVD

The repeatability of RVD obtained by SS-OCTA was excellent for the low-teens NTG patients and healthy subjects (Table 2). The intraclass correlation coefficients (ICCs) for intra-visit repeatability of peripapillary RVD showed excellent reliability (0.835–0.948), as did those for macular RVD (0.863–0.948). The ICCs for inter-visit repeatability of peripapillary RVD ranged from 0.755 to 0.911, and for macular RVD, from 0.806 to 0.898.

**Table 2:** Intra- and inter-visit repeatability of RVD in healthy subjects and low teens-NTG.

	Intra-visit repeatability				Inter-visit repeatability			
	ICC (lower–upper 95% CI)				ICC (lower–upper 95% CI)			
	Total subjects	Healthy subjects	Low teens-NTG	*P*-value*	Total subjects	Healthy subjects	Low teens-NTG	*P*-value*
Peripapillary RVD								
ONH	0.936 (0.836–0.981)	0.924	0.957	0.511	0.755 (0.671–0.947)	0.714	0.827	0.206
pSuperior	0.948 (0.737–0.994)	0.914	0.967	0.456	0.898 (0.867–0.922)	0.865	0.925	0.535
pInferior	0.933 (0.827–0.980)	0.949	0.922	0.627	0.911 (0.705–0.982)	0.963	0.853	0.408
pNasal	0.835 (0.617–0.948)	0.865	0.797	0.225	0.833 (0.637–0.964)	0.842	0.935	0.478
pTemporal	0.874 (0.746–0.986)	0.884	0.870	0.258	0.844 (0.730–0.923)	0.876	0.812	0.400
Macular RVD								
Fovea	0.948 (0.737–0.994)	0.914	0.967	0.456	0.851(0.740–0.927)	0.884	0.821	0.198
mSuperior	0.947 (0.913–0.980)	0.956	0.967	0.598	0.898 (0.786–0.965)	0.914	0.877	0.306
mInferior	0.863 (0.822–0.896)	0.874	0.855	0.478	0.882 (0.704–0.987)	0.866	0.942	0.274
mNasal	0.890 (0.858–0.916)	0.914	0.879	0.104	0.806 (0.673–0.903)	0.832	0.794	0.258
mTemporal	0.907 (0.709–0.981)	0.914	0.899	0.627	0.825 (0.670–0.918)	0.842	0.805	0.188

*As analyzed by repeated-measures ANOVA between Healthy and POAG groups, respectively.

ICC = intraclass correlation coefficient; CI = coefficient interval; RVD = retinal vessel density; ONH = optic nerve head.

### Intra-group assessment of diurnal variations

The RVDs of the peripapillary (ONH, superior, inferior, temporal, nasal) and macular (fovea, superior, inferior, temporal, nasal) areas were assessed in detail (Table 3). The data also was schematized as a scatter plot (Supplementary Fig. 1). In the healthy group, only RVDs of the inferior peripapillary region showed significant diurnal variation (*P* = 0.008), whereas the low-teens NTG group showed sizeable variation in the superior peripapillary, inferior macular, and temporal macular areas (*P* = 0.018, 0.022, and 0.015). Among the hemodynamic parameters, DBP and MOPP of the low-teens NTG group showed significant change on diurnal fluctuation (*P* = 0.002 and 0.017).

**Table 3:** Diurnal distributions of RVD (%) and hemodynamic parameters for each measurement session with ANOVA *P* values.

	Healthy group (N = 32)					Low-teens NTG (N = 35)				
	9AM	12PM	3 PM	6 PM	*P value	9AM	12PM	3 PM	6 PM	*P value
Peripapillary RVD	51.98 ± 3.33	51.87 ± 3.86	52.30 ± 4.25	53.19 ± 3.26	0.856	47.09 ± 5.30	47.71 ± 5.04	47.06 ± 4.86	47.44 ± 5.00	0.532
ONH	38.77 ± 7.98	39.58 ± 8.30	39.88 ± 8.02	40.99 ± 7.20	0.666	30.24 ± 12.37	30.09 ± 12.65	28.80 ± 11.05	32.64 ± 11.21	0.130
pSuperior	61.33 ± 5.82	61.12 ± 5.94	60.51 ± 6.62	61.07 ± 6.21	0.269	56.17 ± 6.62	57.58 ± 9.00	54.17 ± 11.41	55.66 ± 5.80	**0.018**
pInferior	62.59 ± 5.99	61.95 ± 6.66	63.20 ± 7.15	66.06 ± 5.28	**0.008**	55.31 ± 10.16	55.29 ± 9.55	54.99 ± 6.54	55.99 ± 9.28	0.615
pTemporal	49.37 ± 6.46	47.84 ± 7.52	48.72 ± 7.87	49.48 ± 6.41	0.101	48.79 ± 7.06	47.53 ± 7.04	48.42 ± 7.34	46.99 ± 7.06	0.326
pNasal	47.85 ± 5.99	48.86 ± 5.49	49.18 ± 5.91	48.33 ± 6.66	0.782	44.95 ± 11.08	48.03 ± 8.53	48.95 ± 7.26	45.93 ± 8.74	0.732
Macular RVD	40.28 ± 3.35	39.86 ± 2.55	40.78 ± 3.51	40.80 ± 2.89	0.205	40.17 ± 3.32	41.03 ± 3.54	40.99 ± 3.27	40.72 ± 2.66	0.582
Fovea	14.47 ± 5.28	14.91 ± 4.80	15.72 ± 7.99	14.38 ± 4.45	0.514	14.62 ± 4.31	15.60 ± 6.37	15.63 ± 5.51	14.33 ± 4.02	0.647
mSuperior	48.27 ± 6.18	47.28 ± 4.09	47.92 ± 4.23	49.16 ± 4.38	0.455	48.62 ± 5.48	50.20 ± 5.47	48.24 ± 4.97	49.66 ± 4.13	0.184
mInferior	49.92 ± 5.52	47.82 ± 4.75	50.68 ± 6.06	48.32 ± 4.60	0.124	45.36 ± 7.56	47.75 ± 6.58	48.61 ± 6.15	47.34 ± 6.70	**0.022**
mTemporal	45.59 ± 5.25	45.58 ± 3.43	45.69 ± 4.08	47.44 ± 4.35	0.534	46.72 ± 4.71	45.96 ± 5.12	46.04 ± 4.03	45.50 ± 5.26	**0.015**
mNasal	43.16 ± 5.87	43.73 ± 3.99	44.11 ± 5.11	44.69 ± 4.62	0.949	45.52 ± 8.71	45.65 ± 5.78	46.41 ± 5.56	46.75 ± 5.39	0.484
SBP (mmHg)	125.21 ± 13.78	125.79 ± 11.74	125.86 ± 11.66	126.24 ± 12.53	0.743	131.06 ± 15.74	126.09 ± 15.28	128.53 ± 13.85	125.94 ± 23.58	0.294
DBP (mmHg)	78.79 ± 11.38	78.29 ± 10.75	78.70 ± 9.98	79.33 ± 9.57	0.691	78.29 ± 11.01	75.79 ± 10.56	76.35 ± 7.98	69.79 ± 10.04	**0.002**
IOP (mmHg)	14.55 ± 2.06	14.05 ± 2.53	13.88 ± 2.29	14.37 ± 2.50	0.476	14.03 ± 1.87	13.71 ± 1.66	13.71 ± 1.53	14.56 ± 2.13	0.163
MOPP (mmHg)	53.47 ± 8.05	53.02 ± 7.03	53.84 ± 7.03	54.16 ± 6.85	0.575	53.22 ± 7.19	52.39 ± 6.15	52.93 ± 5.65	50.06 ± 6.90	**0.017**

* Repeated-measures ANOVA; bolded values represent significance, P < 0.05.

RVD = retinal vessel density; ONH = optic nerve head; SBP = systolic blood pressure; DBP = diastolic blood pressure; IOP = intraocular pressure; MOPP = mean ocular perfusion pressure.

### Magnitudes of diurnal variations

To determine the degree of diurnal variation, the maximal change (maximum—minimum values among 4 measurements) of RVD and hemodynamic parameters was analyzed (Table 4). The magnitudes of diurnal variation of average peripapillary and macular RVD variations in low-teens NTG (3.85 ± 2.83 and 4.92 ± 3.83%) were more variable than in the healthy group (2.64 ± 1.55 and 3.38 ± 2.43%) (*P* = 0.017 and 0.036). In detail, the RVD’s diurnal change of ONH (6.83 ± 3.90 vs 10.43 ± 8.57, *P* = 0.005) and superior peripapillary (5.61 ± 3.29 vs 9.85 ± 10.44, *P* = 0.015), inferior peripapillary (7.03 ± 4.84 vs 11.68 ± 9.67, *P* = 0.008), and nasal macular (5.09 ± 3.93 vs 8.38 ± 6.49, *P* = 0.007) areas in the low-teens NTG eyes were larger than in the healthy eyes. Similarly, among the hemodynamic variables, the maximal changes of DBP (12.65 ± 7.17vs 16.90 ± 7.35, *P* = 0.013) and MOPP (7.14 ± 4.43 vs 9.57 ± 5.27, *P* = 0.025) in low-teens NTG were greater than those in the healthy group.

**Table 4:** Magnitude of diurnal variation of RVD and hemodynamic parameters between healthy subjects and low-teens NTG.

	Healthy group (N = 32)	Low-teens NTG (N = 35)	*P*-value*
ΔMax.in peripapillary RVD (%)			
Average	2.64 ± 1.55	3.85 ± 2.83	**0.017**
ONH	6.83 ± 3.90	10.43 ± 8.57	**0.005**
pSuperior	5.61 ± 3.29	9.85 ± 10.44	**0.015**
pInferior	7.03 ± 4.84	11.68 ± 9.67	**0.008**
pTemporal	6.72 ± 4.89	8.07 ± 4.38	0.216
pNasal	5.92 ± 3.92	8.90 ± 9.01	0.080
ΔMax.in macular RVD (%)			
Average	3.38 ± 2.43	4.92 ± 3.83	**0.036**
Fovea	5.54 ± 7.22	4.44 ± 4.87	0.450
mSuperior	6.18 ± 4.30	7.13 ± 4.41	0.347
mInferior	7.35 ± 5.80	7.90 ± 4.78	0.658
mTemporal	5.59 ± 4.40	5.46 ± 5.49	0.910
mNasal	5.09 ± 3.93	8.38 ± 6.49	**0.007**
ΔMax.in hemodynamics (mmHg)			
SBP	16.38 ± 7.90	20.43 ± 19.20	0.218
DBP	12.65 ± 7.17	16.90 ± 7.35	**0.013**
IOP	2.69 ± 1.46	2.71 ± 1.38	0.963
MOPP	7.14 ± 4.43	9.57 ± 5.27	**0.025**

*Independent t-test, bolded values represent significance, *P* < 0.05.

RVD = retinal vessel density; ONH = optic nerve head; SBP = systolic blood pressure; DBP = diastolic blood pressure; IOP = intraocular pressure; MOPP = mean ocular perfusion pressure.

### Patterns of diurnal variations

**Figure 1:**
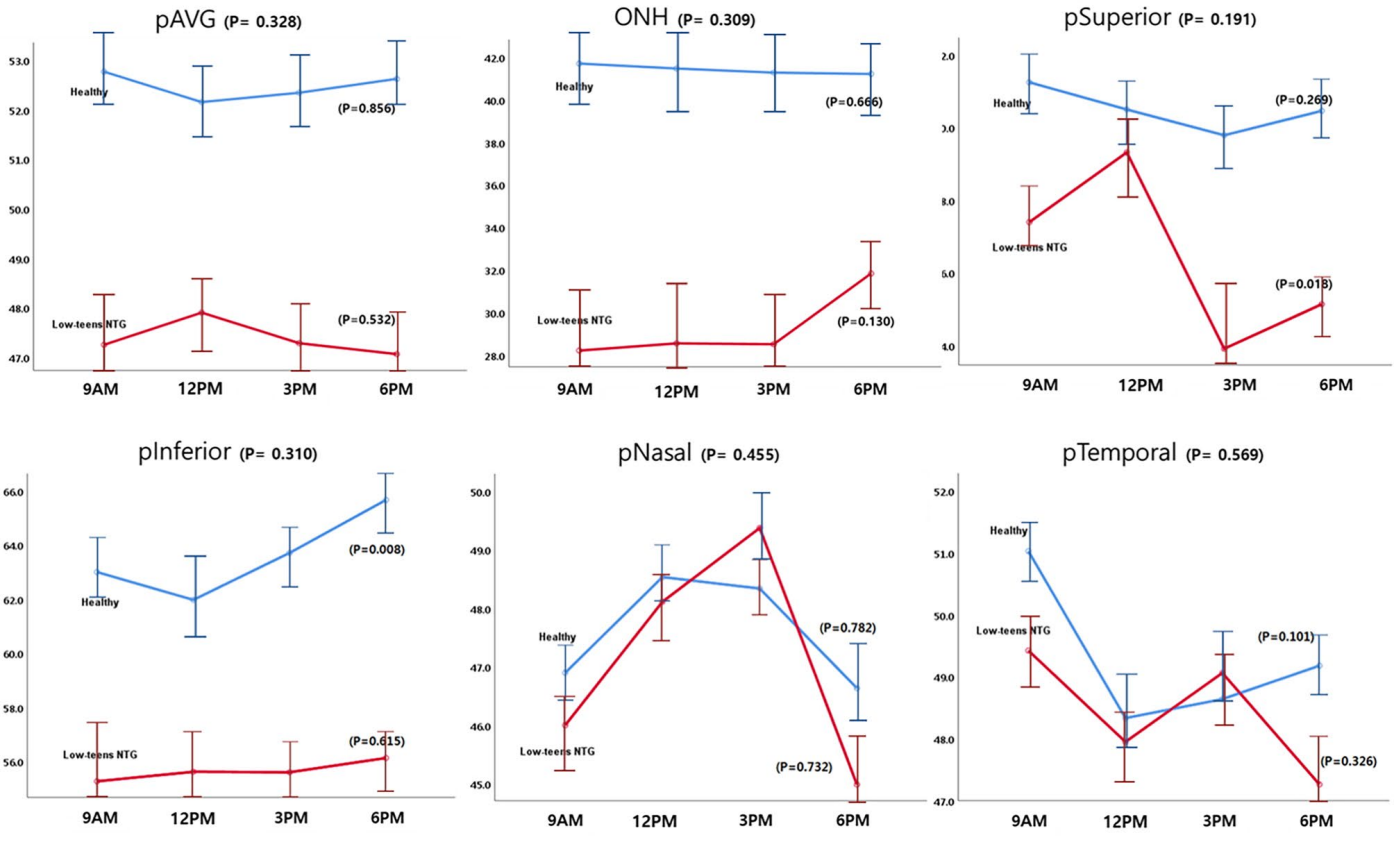
Patterns of diurnal variation of peripapillary RVD in healthy subjects and low-teens NTG patients. The average RVDs in the peripapillary (ONH, superior, inferior, temporal, nasal) areas are shown at the top left. The RVDs in each group were assessed by ANOVA, and the inter-group comparison was analyzed by linear mixed model.

**Figure 2:**
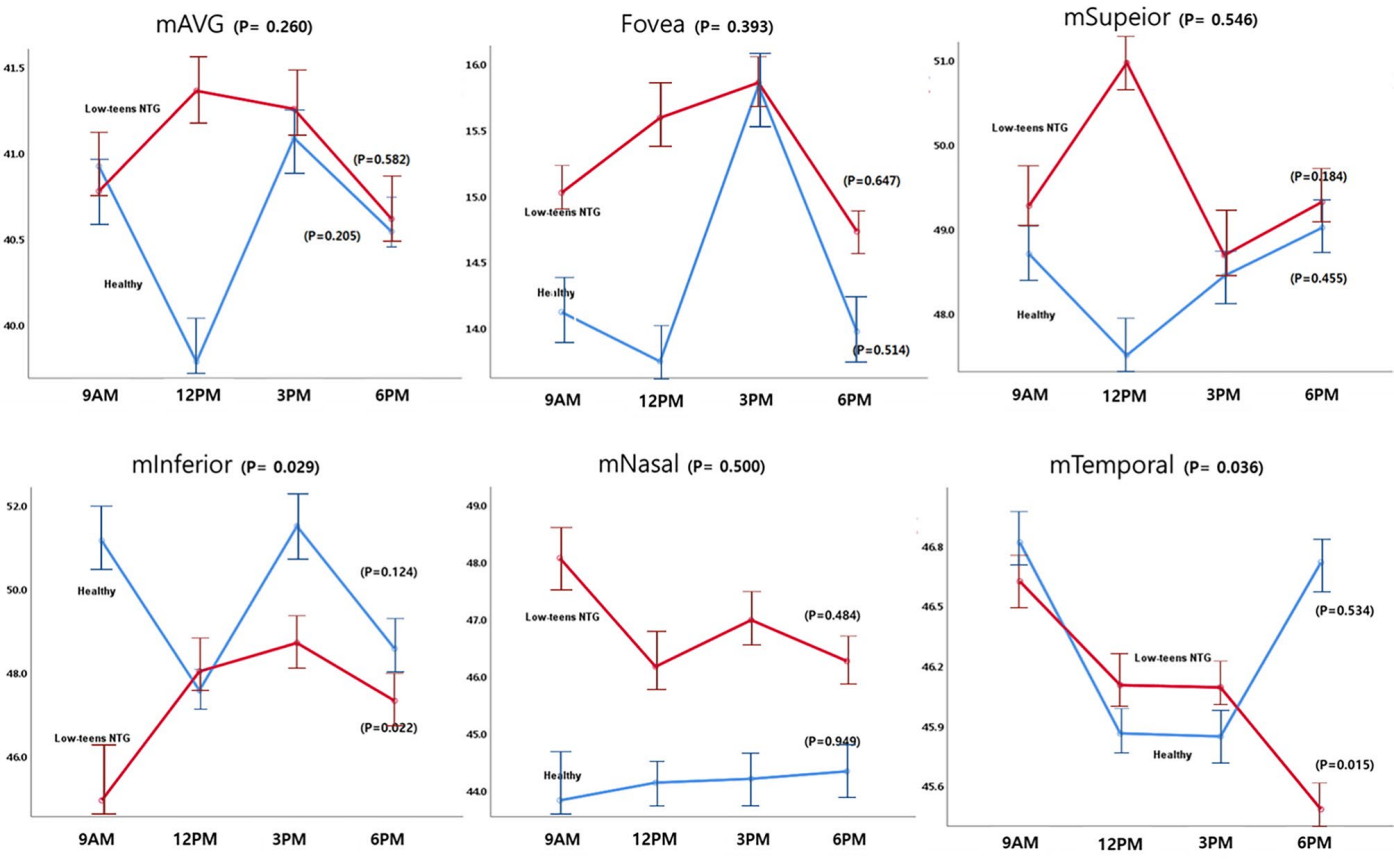
Patterns of diurnal variation of macular RVD in healthy subjects and low-teens NTG patients. The average RVDs in the macular (fovea, superior, inferior, temporal, nasal) areas are shown at the top left. The RVDs in each group and inter-group comparison were analyzed, respectively.

**Figure 3:**
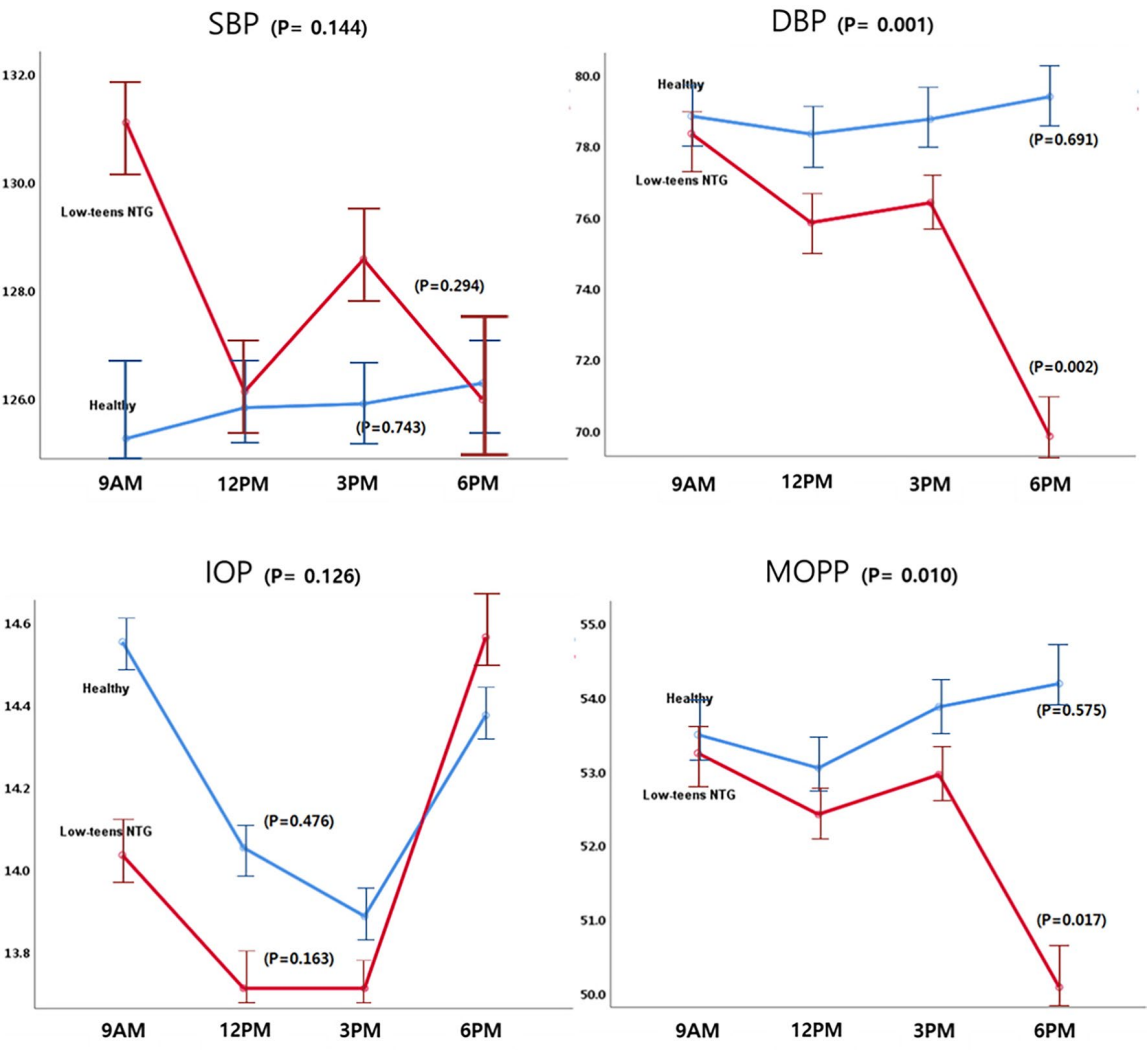
Patterns of diurnal variation of hemodynamic parameters in healthy subjects and low-teens NTG patients. SBP, DBP, IOP, and MOPP were recorded every 3 h from 9 a.m. to 6 p.m. Intra-group and inter-group analyses for the hemodynamic variations were assessed, respectively.

The patterns of diurnal changes of peripapillary RVD (Fig. 1), macular RVD (Fig. 2), and hemodynamic parameters (Fig. 3) between the two groups were examined, and the intra- and inter-group variabilities were assessed. The diurnal RVD of the peripapillary areas did not show any significant pattern difference between the two groups (all *P* > 0.05, Fig. 1). However, a comparative analysis of the diurnal macular RVD changes between the two groups found differences in some sectors. Specifically, the inferior and temporal macular RVD showed different patterns in the inter-group analysis (*P* = 0.029 and 0.036, Fig. 2). As for the hemodynamic parameters, the low-teens NTG patients and healthy subjects showed different patterns of diurnal variation in DBP and MOPP (*P* = 0.001 and 0.010, Fig. 3).

### Post hoc analysis of pattern changes

For the variables showing inter-group differences, a post hoc analysis was performed to confirm which data-acquisition session(s) were most contributory (Table 5). The inferior macular RVD of the healthy group was considerably higher than that of the low-teens NTG at 9 a.m.(P = 0.005), and temporal macular RVD was higher at 6 p.m.(P = 0.023). As for the hemodynamic parameters, the DBP and MOPP of the low-teens NTG group had trough values at 6 p.m., whereas the healthy group showed its peak DBP and MOPP values in that session (*P* =  < 0.001 and 0.012).

**Table 5:** Post hoc analysis of macular RVD, DBP, and MOPP in healthy group and low teens-NTG.

	Healthy group (N = 32)	Low-teens NTG (N = 35)	*P*-value*
Inf. macular RVD (%)			
9 AM	49.92 ± 5.52	45.36 ± 7.56	**0.005**
12 PM	47.82 ± 4.75	47.75 ± 6.58	0.956
3 PM	50.68 ± 6.06	48.61 ± 6.15	0.171
6 PM	48.32 ± 4.60	47.34 ± 6.70	0.507
Temp. macular RVD (%)			
9 AM	45.59 ± 5.25	46.72 ± 4.71	0.347
12 PM	45.58 ± 3.43	45.96 ± 5.12	0.703
3 PM	45.69 ± 4.08	46.04 ± 4.03	0.728
6 PM	47.44 ± 4.35	45.50 ± 5.26	**0.023**
DBP (mmHg)			
9 AM	78.79 ± 11.38	78.29 ± 11.01	0.849
12 PM	78.29 ± 10.75	75.79 ± 10.56	0.314
3 PM	78.70 ± 9.98	76.35 ± 7.98	0.258
6 PM	79.33 ± 9.57	69.79 ± 10.04	** < 0.001**
MOPP (mmHg)			
9 AM	53.47 ± 8.05	53.22 ± 7.19	0.888
12 PM	53.02 ± 7.03	52.39 ± 6.15	0.684
3 PM	53.84 ± 7.03	52.93 ± 5.65	0.541
6 PM	54.16 ± 6.85	50.06 ± 6.90	**0.012**

*Independent t-test; bolded values represent significance, *P* < 0.05.

RVD = retinal vessel density; DBP = diastolic blood pressure; MOPP = mean ocular perfusion pressure.

## Discussion

This study was a comparative analysis of the magnitude and pattern of diurnal RVD variation between low-teens NTG patients and healthy eyes. We found that the magnitude of diurnal RVD variation was more significant in low-teens NTG. As for the pattern of diurnal variation, macular RVD showed different patterns between the two groups. Characteristically, both DBP and MOPP also showed higher diurnal variation in the low-teens NTG group, with a descending pattern in the evening.

The present study has some strengths in its design. First, we studied exclusively low-teens NTG patients, who would be expected to be more vulnerable to hemodynamic variability compared with patients experiencing glaucoma with higher IOPs. Second, only treatment-naïve patients were studied; thus, we could exclude the effect of IOP-lowering medication on diurnal fluctuation. Third, IOP, RVD and systemic BP were simultaneously measured through a prospective analysis.

As mentioned in the introduction, the authors have already reported on the diurnal variation of RVD, IOP, and BP in POAG patients^8^. An earlier study of ours demonstrated that diurnal IOP variation in low-teens NTG is an independent risk factor for glaucoma progression^4^. According to multivariate regression analyses, the significant risk factors for progression in low-teens NTG were fluctuation of DBP (HR = 1.058), diurnal IOP fluctuation (HR = 1.609), and occurrence of disc hemorrhage (HR = 3.661). According to these findings, diurnal variation of BP, in addition to IOP, is an important parameter to evaluate in cases of low-teens NTG. However, these patients with POAG mostly had high baseline IOP, and thus, mechanical damage from high IOP rather than hemodynamic factors might have been the dominant mechanism of GON. Also, because the participants had been using glaucoma medication, our previous studies may not have fully represented the actual hemodynamic variability. In this respect, this study was conducted to supplement the previous research, and the diurnal variation of RVD and MOPP before treatment were analyzed in low-teens NTG.

Notably, low ocular perfusion pressure (OPP) can occur as a result of low BP, even with normal IOP (< 21 mmHg). Also, in terms of OPP fluctuation, OPP sometimes is more sensitive to BP changes than to IOP changes, because BP varies in the range of 40 to 60 mmHg while IOP varies in the range of 5 to 8 mmHg^10^. In this regard, several epidemiological studies have identified low systolic BP (SBP) and DBP as an important risk factor for development and progression of glaucoma^11–14^. Some studies have also identified nocturnal BP dips as a risk factor for glaucoma progression^15,16^.

In particular, the DBP behavior of low-teens NTG in this study was distinct: it appeared at the same level as the healthy controls in the morning and afternoon (from 9 a.m. to 3 p.m.), but became significantly lower during the evening (6 p.m.), which coincided with the time during which the MOPP was lower. This would explain why MOPP was consistently lower in low-teens NTG in the evening. The association between DBP reduction and lower MOPP in low-teens NTG may be attributable to dysregulation of the autonomic nervous system^17^. A longitudinal study of chronic hypotension found that low BP cases were associated with low BMI, heart rate and body temperature, reflecting the lowered sympathetic nervous system in hypotension^18^.

In this study, we focused on not only MOPP level but also MOPP variability. The degrees of diurnal change of macular RVD accompanied by DBP and MOPP change were greater in the low-teens NTG group than in the healthy subjects. Previous studies have identified larger variability of MOPP as a risk factor for glaucoma progression^19,20^. Similarly, the present study observed significant variations of systemic hemodynamics and ocular hemodynamic changes simultaneously at the level of the retinal blood vessels in the low-teens NTG setting.

Under normal physiological conditions, low perfusion pressure is compensated by low resistivity to flow^21^. One possible explanation for this phenomenon is the effect of compensation mechanisms, which is to say, autoregulation. In healthy individuals, significant fluctuations in retinal circulation did not occur, as autoregulation to compensate for variation of MOPP works properly. Another OCTA study of ours on the systemic BP and IOP of 132 healthy participants (264 eyes) under 45 years of age demonstrated that peripapillary RVD was not correlated with MOPP^22^. Considering the young age and disease-free state of our study population, autoregulation most likely was at work. On the other hand, it could also be deduced that this compensation mechanism does not work properly in low-teens NTG groups^19,20^. If OPP is not constantly maintained and fluctuates markedly, oxidative stress on the ONH can result. OPP fluctuation can cause ischemia–reperfusion damage to retinal ganglion cells (RGCs) by oxygen-free radicals and nitric oxide species^23^. Additionally, OPP that fluctuates below the lower autoregulatory limit is more susceptible to RGC damage and ONH ischemia^24^.

Verticchio Vercellin AC et al.^25^ evaluated diurnal variations in peripapillary RVD as assessed by OCTA in healthy subjects as well as ocular hypertension and OAG patients. The results demonstrated that the variations in the OCTA-derived parameters were relatively small among the three groups. This discrepancy from our results appears to have been due to a difference of study design. Our study was conducted on low-teens NTG in the treatment-naïve state, whereas Verticchio Vercellin AC's study had evaluated diurnal variation in glaucoma patients being administered ocular hypotensive medication. According to previous reports, ocular hypotensive medication reduces diurnal fluctuation^26,27^. Therefore, the actual severity of diurnal RVD variation might have been underestimated in that earlier study.

In this study, significant diurnal variation of RVD was observed in the superior peripapillary sector, inferior macular sector, and temporal macular sector of low-teens NTG patients. There are some possible explanations for the sectoral differences in RVD variability seen in NTG eyes. The RNFL thickness in the superior and inferior peripapillary areas was relatively thicker than in the nasal and temporal areas, and the RVD was higher^28^. In addition, circumpapillary retinal vessel RVD was correlated with RNFLthickness^29^. That is, there is a possibility that the diurnal variation was measured larger in that area because the initial value was thicker. The next hypothesis that can be considered is that initial RNFL damage occurs in the superotemporal or inferotermpoal area, and especially the interotemporal area connecting to the macula is known as a macular-vulnerable zone. This zone is part of the high-density axon region known to be vulnerable to glaucomatous damage^30^. A follow-up study that topographically analyzes whether these glaucoma-damage-prone areas are correlated with higher variation of RVD seems necessary.

Interestingly, the RVD in the inferior region of the macula was lowest in the morning (9:00AM), not in the evening (6:00PM). Although a clear mechanism could not be delineated, the authors considered two possible answers. The standard deviation of the inferior macular RVD value at 9:00 am was relatively larger (± 7.56). And since this study had a relatively small sample size, we assumed that some outliers had made for low RVD at these points. Next, the 6 PM test time specified in this study may not be sufficient to reflect the actual rhythm of RVD change. The RVD value might decrease further after 6 PM, but in this study, the OCTA scans were conducted in the early evening (6 PM).

Several limitations need to be considered when interpreting the results of this study. First, RVD and hemodynamics were not assessed at midnight. Uniformly across the entire diurnal cycle, notably, it was not practical to measure patients at the midnight time point. Thus, the significant pattern of RVD variation that we observed might not reflect the whole diurnal variation. Also, changes in IOP, BP, and RVD according to postural change were not considered in this study. In the near future, additional assessments are needed to analyze RVD based on 24-h monitoring of BP and IOP according to postural change. Second, another possible limitation that may have influenced diagnostic validity is ‘white coat syndrome,’^31^ which refers to stress on study participants in cases where their BP measurements are obtained by clinicians, which readings might be rendered inaccurate as a result. Third, MOPP, as an estimate of OPP, might differ from actual OPP at the ONH, for which reason brachial-arterial pressure has been used as a surrogate, albeit not an ideal one, for ophthalmic arterial pressure^22^. Fourth, and finally, diurnal variations might be more or less pronounced, or have a different rhythm, in NTG. It might simply be that the rhythm could not be aligned with the specific times tested between the groups. Additional measurements for all subjects and on consecutive days would be helpful in clarifying this point.

In conclusion, in low-teens NTG eyes, diurnal changes of RVD, DBP and MOPP were greater than in the healthy eyes, and the patterns of diurnal variation significantly differed. Our findings suggest that diurnal RVD variation might reflect the hemodynamic variation characteristic of low-teens NTG. It is assumed that the diurnal variation of systemic BP and MOPP rather than of IOP will act as an important factor in diurnal RVD variation. Therefore, in addition to assessment of IOP fluctuation in low-teens NTG patients, diurnal variation of BP and MOPP should be considered when evaluating progressive low-teens NTG patients. In the future, complementary studies should be conducted to determine whether diurnal variation of RVD shows a significant association with functional and structural data and whether it can be a risk factor for glaucoma progression in NTG patients.

## Methods

### Ethics

This study was approved by the Institutional Review Board of Hallym University Sacred Heart Hospital, and all of the protocols were in accordance with the tenets of the Declaration of Helsinki. Each subject was required to sign an informed consent statement before being enrolled in the study and prior to any study measurements.

### Study subjects

This was a prospective cohort study of low-teens NTG and age-matched healthy subjects. All of the subjects were prospectively enrolled at the Glaucoma Clinic of Hallym Sacred Heart Hospital between November 2019 and July 2021.

Low-teens IOP was defined as mean untreated office IOP < 15 mmHg in diurnal measurements (at 3-h intervals from 9:00 a.m. to 6:00 p.m., for a total of 4 measurements). Low-teens NTG patients who met the following inclusion criteria were initially enrolled: (1) treatment-naïve status (diurnal variation analysis prior to anti-glaucoma medication administration), and (2) normal open-angle on gonioscopy. All of the participants had to meet the following further criteria for inclusion: age > 18 years, best-corrected visual acuity (BCVA) ≥ 20/40, and spherical equivalent < -6.0 diopters. Patients with a history of ocular surgery except uncomplicated cataract surgery, or any history of retinal pathology or non-GON were excluded. Participants also were excluded in cases of diagnosis of neurodegenerative disease or cerebrovascular accident history. After evaluating the enrollment of low-teens NTG group, we then consecutively recruited healthy volunteers from a comprehensive ophthalmology clinic in order to match the age distribution of the glaucoma patients.

Participants with diabetes mellitus and systemic hypertension were included unless they had been diagnosed with diabetic or hypertensive retinopathy. Antihypertensive medications were recorded on the basis of the number of medications taken and the type of medication (angiotensin-converting enzyme inhibitors[ACEIs], angiotensin II receptor blockers, calcium channel blockers [CCBs],diuretics or beta receptor blockers [BRBs])^32^.

All of the participants underwent complete ophthalmologic examinations, including baseline IOP measurement by Goldman applanation tonometry, BCVA, refractive value with an autorefractor (HRK-8000A, Huvitz, Gunpo, Korea), slit-lamp examination, gonioscopy, and dilated fundus examination. After pupil dilation, all of the participants were imaged by stereo optic disc photography and red-free (RNFL photography (Vx-10; Kowa Optimed Inc., Tokyo, Japan). They also undertook standard automated perimetry using the Swedish interactive threshold algorithm with the 24–2 standard program (Humphrey Field Analyzer II; Carl Zeiss Meditec Inc., Dublin, CA, USA).

Glaucomatous eyes were defined as those showing glaucomatous optic disc neuropathy (e.g., notching, neuroretinal rim thinning, and/or RNFL defects) and corresponding glaucomatous visual field defects, as confirmed by at least two consecutive visual field examinations. The glaucomatous visual field defects were defined as a cluster of ≥ 3 points with P < 0.05 on the pattern deviation map in at least one hemifield, including ≥ 1 point with P < 0.01; a pattern standard deviation (PSD) of P < 0.05; or glaucoma hemifield test result outside the normal limits. Glaucoma clinical severity was assessed for each eye based on mean deviation (MD) thresholds from the modified Hodapp-Anderson-Parish classification scheme, as follows: early (MD ≥  − 6.00 dB), moderate (− 6.01 to − 12.00 dB), advanced (− 12.01 to − 20.00 dB), and severe (− 20.00 dB) glaucoma stages, respectively^33^.

### Assessment of diurnal variation of RVD by swept-source optical coherence tomography angiography (SS-OCTA)

The OCTA scans were performed at four different time points over a single day or on two consecutive days, at 3-h intervals: 9:00 a.m., 12:00, 3:00, and 6:00 p.m. All of the scans were performed by a single operator (Supplementary Fig. 2).

In this study, we applied a mode of swept-source OCTA (SS-OCTA; DRI OCT Triton, Topcon, Tokyo, Japan) to visualize the retinal vasculature of the peripapillary area and macula. The scans were taken from a 4.5 × 4.5 mm cube, each consisting of 320 clusters of four repeated B-scans centered on the ONH and fovea. Slabs of retinal capillary plexus were segmented by the built-in software (IMAGEnet6, v1.23.15008, Basic License 10). Only the superficial capillary plexus (SCP) was adopted for analysis. The SCP was delineated from 2.6 μm below internal limiting membrane to 15.6 μm below the junction between inner plexiform and inner nuclear layers. The DRI OCT Triton characterizes vascular information quantitatively by vessel density (VD; %) as well as qualitatively at various user-defined retinal layers on a vessel density (VD) map. RVD was automatically calculated as the proportion of the area occupied by flowing blood vessels defined as pixels having above-threshold-level decorrelation values acquired by the OCTARATM (OCTA Ratio Analysis) algorithm^34^. Peripapillary and macular RVD were measured at the optic disc and fovea and at each of the adjacent four sites employing a modified version of the Early Treatment Diabetic Retinopathy Study (ETDRS) grid (Supplementary Fig. 3).

### Assessment of oculo- and hemodynamic parameters

IOP and BP were also measured four times, every 3 h from 9:00 a.m. to 6:00 p.m. over a single day or on two consecutive days (Supplementary Fig. 2). That is, there were four measurement sessions (as specified above), and SS-OCTA, IOP, and BP were all taken for each session. To exclude any inter-measurement effects, all patients’ examinations were taken in the order of systemic BP, IOP, and SS-OCTA. In addition, to minimize the residual effect of accommodation or pupillary dilation during the examination, each test interval was more than 5 min apart.

The IOPs of the recruited eyes were measured by Goldmann applanation tonometry in every session. All of the participants were in a sitting position and told to relax before measurement. IOP was measured three times for each session, and the median value was adopted. A single investigator (S.U.B.) performed all of the IOP measurements. The systemic BP including SBP and DBP was measured at the upper arm with an automated sphygmomanometer (OMRON HBP-1300, OMRON Healthcare, Co., Ltd China.) while the patient was asked to remain calm in a sitting position for at least 5 min.

According to the measured IOP and BP data for each session, the mean arterial pressure (MAP) and MOPP were analyzed. The MAP was calculated as follows: MAP = DBP + [1⁄3 × (SBP − DBP)]^35,36^. It was thus possible to calculate the MOPP at any specified time from the difference between the MAP and the IOP (substituted for venous pressure) as follows: MOPP = 2⁄3 × (MAP − IOP).

### Intra- and Inter-visit repeatability of RVD measurements

Repeatability of RVD was evaluated for the first 10 individuals of each group. The intra-visit repeatability of the peripapillary and macular RVDs was calculated from a subset of 10 healthy eyes and 10 low-teens NTG eyes, three sets of scans having been performed on a single visit. The ICCs, as obtained from three sets of scans performed on three separate visits, were used to evaluate test–retest variability^37^. The inter-visit reproducibility of the peripapillary and macular RVDs was analyzed from the same healthy subjects and low-teens NTG patients. Three OCT scans were performed within 30 min on 3 different days. The dataset measured for reliability was used independently from the dataset used for diurnal variation evaluation.

### Statistical analysis

The sample size was determined by referring to existing studies^38,39^. The sample-size calculation was based on the assumption of a 10% difference in variation of RVD between the low-teens NTG and healthy groups with a 1-to-1 ratio of glaucomatous to healthy eyes. For a power of 90%, the required sample size was established to be 30 individuals per group.

Test–retest variability was evaluated by ICCs obtained with a two-way random model. The ICC values ranged from 0 to 1, a higher value indicating better reliability. ICCs < 0.40 were considered poor, those between 0.4 and 0.6 fair, those between 0.61 and 0.8 good, and those greater than 0.8, excellent^40^. Repeated-measures analysis of variance (ANOVA) was used to evaluate diurnal changes in RVD, IOP, BP, and MOPP in the two groups. Additionally, to investigate inter-group differences in diurnal changes of RVD, we ran a linear mixed model with adjusted ophthalmological and systemic factors followed by post hoc testing using the independent t-test. All other statistical analyses were performed using the Statistical Package for the Social Sciences version 22.0 for Windows (IBM Corp., Armonk, NY, USA). Statistical significance was defined as P < 0.05.

## Supplementary Information

Supplementary Information.

## Data Availability

Data supporting the findings of the current study are available from the corresponding author on reasonable request.
