# Supplementary material for: Diurnal change of retinal vessel density related to hemodynamic variation in treatment-naïve low-teens normal-tension glaucoma
Source: Sci Rep. 2023 Jun 30;13:10613. doi: 10.1038/s41598-023-37214-w (PMC10313821; doi:10.1038/s41598-023-37214-w)

Supplementary Content

Supplementary Figure 1. Scatterplot of diurnal distributions of RVD (%) and hemodynamic parameters (mmHg)

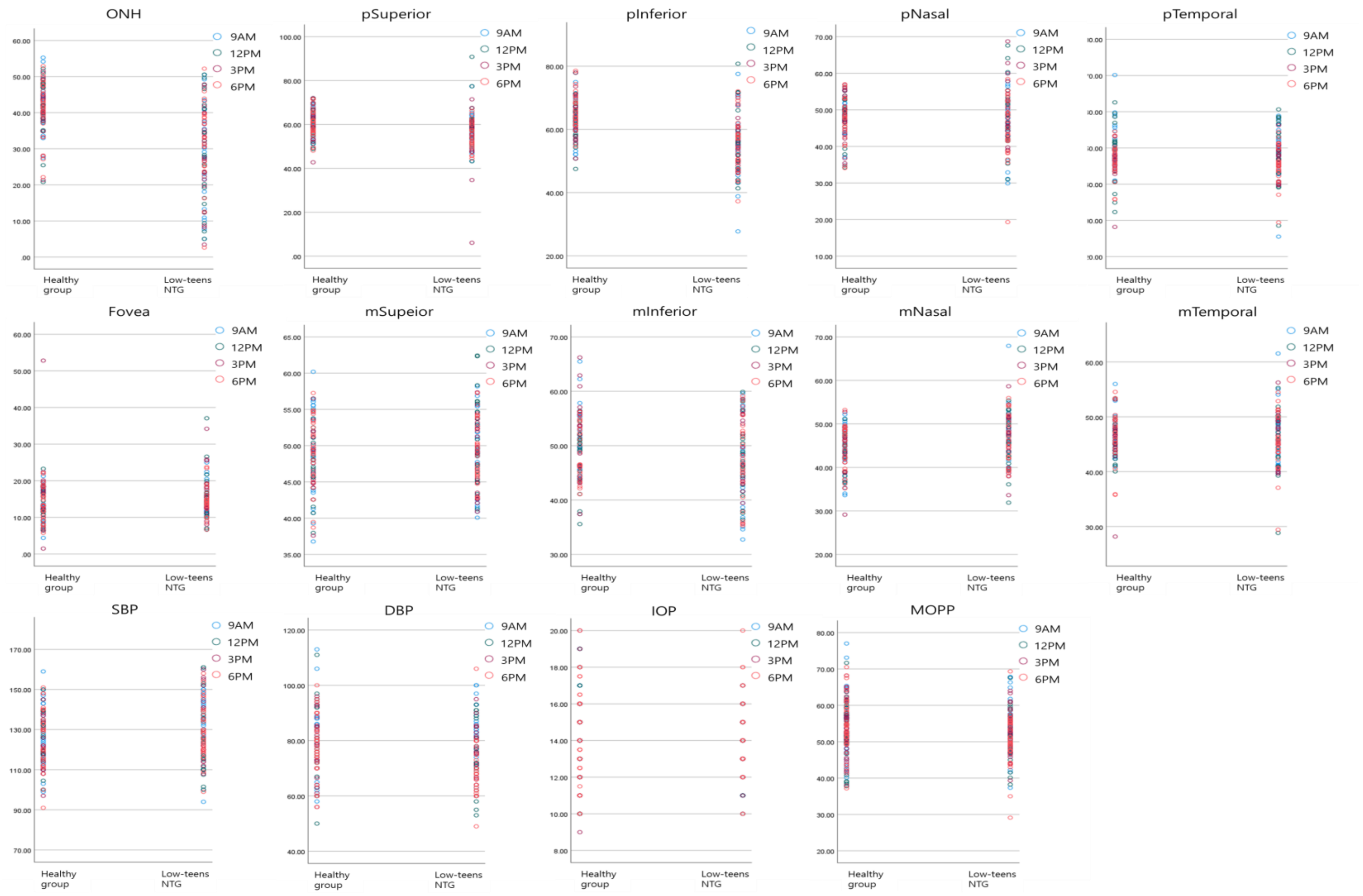

Supplementary Figure 2. Schematic representation of study protocol

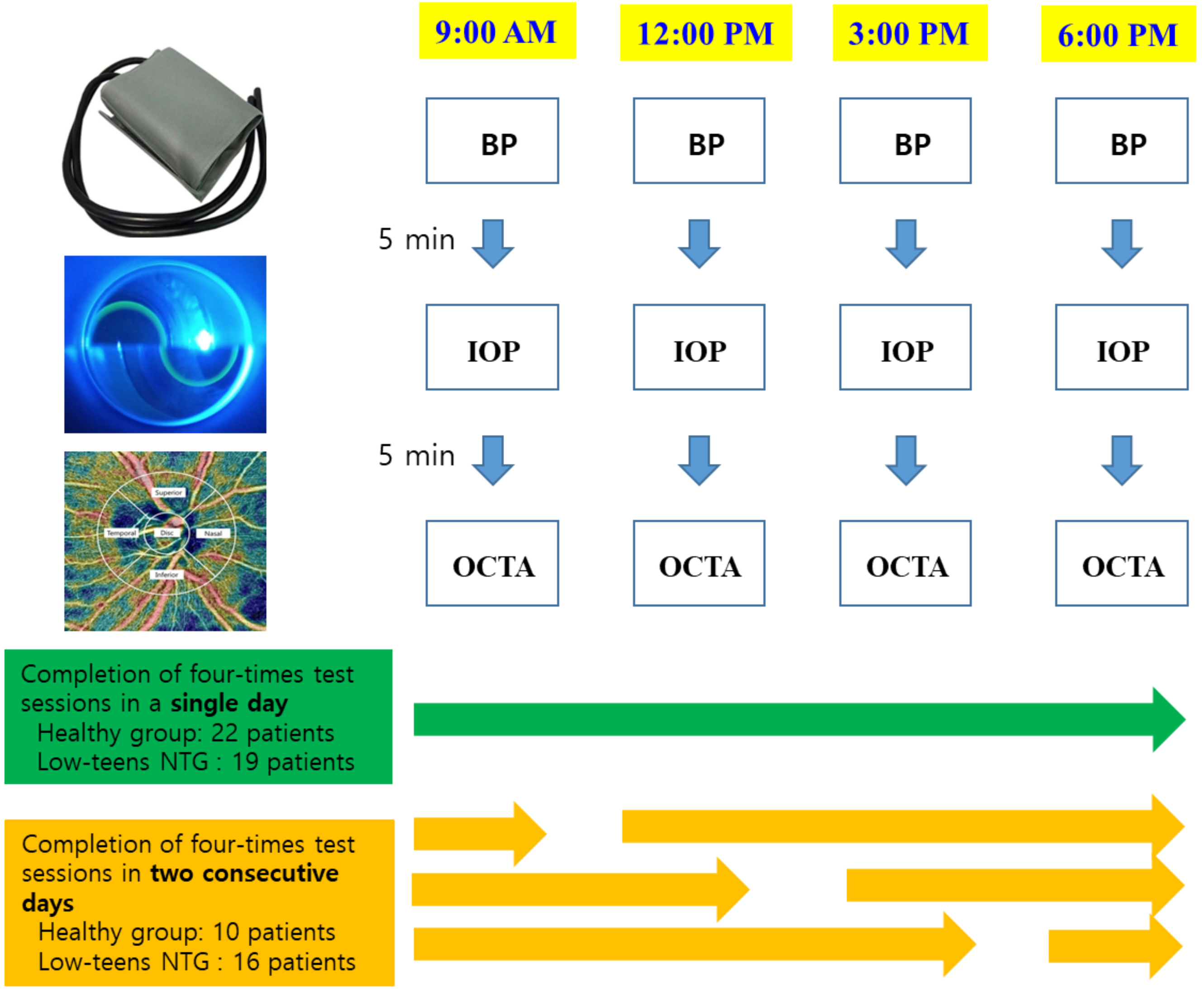

Supplementary Figure 3. Swept-source optical coherent tomography angiography (SS-OCTA) scan of (A) peripapillary and (B) macula area.

This 4.5 x 4.5 mm scan shows superficial vascular plexus as vessel density (VD), generated according to brightneess averages of OCTA images. The modified ETDRS grid consists of two circles, an outer circle of 3.0 mm diameter and an inner circle of 1.0 mm diameter from the centers of the optic disc head and fovea, respectively. Additionally, a central inner circle (0.785 mm^2) was defined as the optic nerve head (ONH) and fovea, and each parafoveal and circumpapillary region was defined as a ring surrounding the circular sector. The parafoveal and circumpapillary regions also were divided into 4 sectors of 90 degrees, namely superior, inferior, temporal and nasal (1.571 mm^2, respectively).

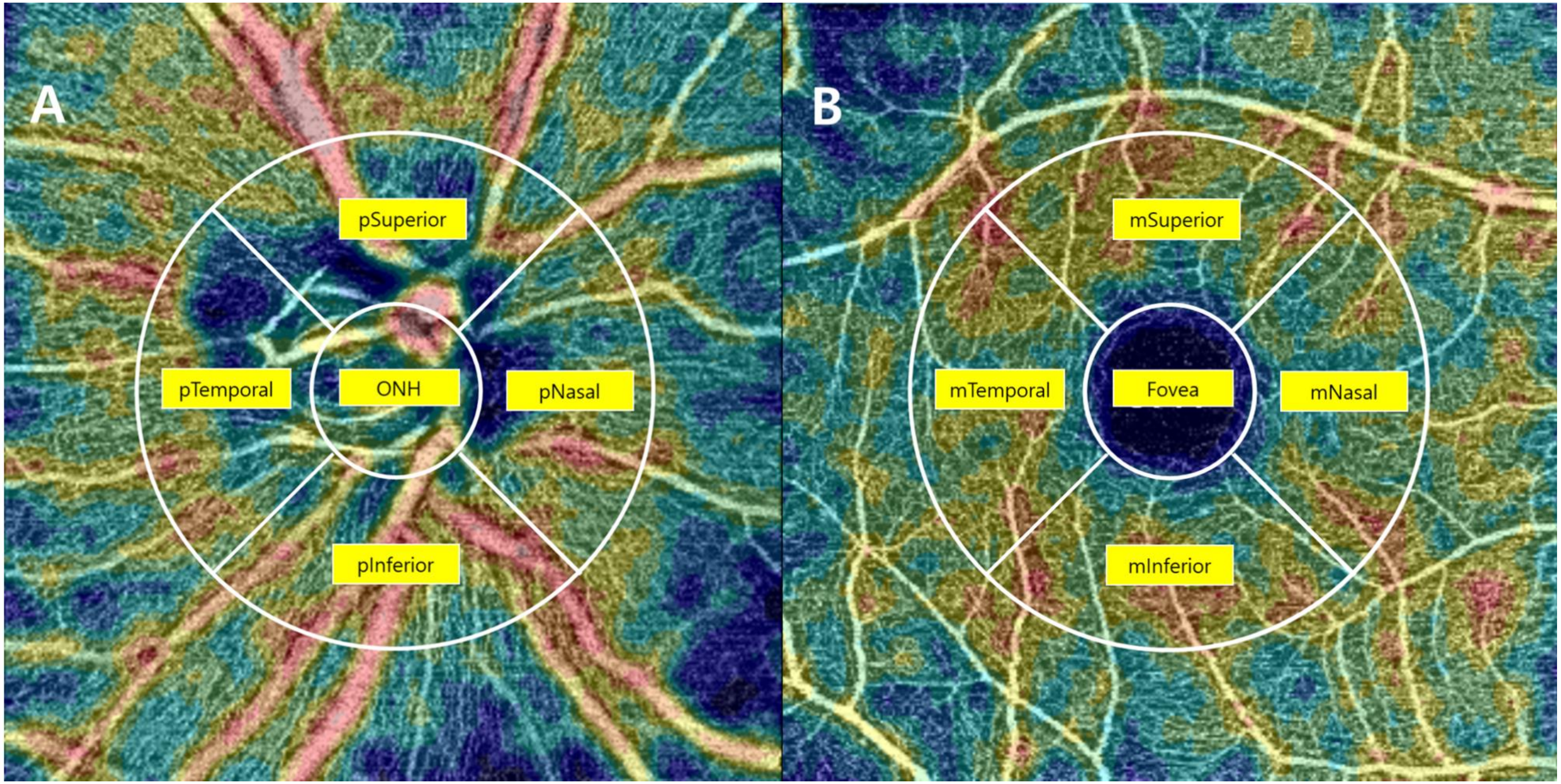

Supplement: Supplementary file 1 — Supplementary Information. [file 41598_2023_37214_MOESM1_ESM.pdf]
